# Differences in clinical presentation, severity, and treatment of COVID-19 among individuals with Down syndrome from India and high-income countries: Data from the Trisomy 21 Research Society survey

**DOI:** 10.7189/jogh.12.05035

**Published:** 2022-08-08

**Authors:** Halder Pinku, Anke Hüls, Patrick T Feany, Nicole Baumer, Mara Dierssen, Stefania Bargagna, Alberto CS Costa, Brian A Chicoine, Anne-Sophie Rebillat, Giuseppina Sgandurra, Diletta Valentini, R Tilman Rohrer, Johannes Levin, Monica Lakhanpaul, Angelo Carfì, Stephanie L Sherman, Andre Strydom, Sujay Ghosh, the Trisomy 21 Research Society COVID-19 Initiative Study Group

**Affiliations:** 1 Cytogenetics and Genomics Research Unit, Department of Zoology, University of Calcutta, Kolkata, West Bengal, India; 2 Department of Epidemiology, Rollins School of Public Health, Emory University, Atlanta, Georgia, USA; 3 Gangarosa Department of Environmental Health, Rollins School of Public Health, Emory University, Atlanta, Georgia, USA; 4 Boston Children’s Hospital and Harvard Medical School, Boston, Massachusetts, USA; 5 Centre for Genomic Regulation (CRG), The Barcelona Institute of Science and Technology, Barcelona, Spain; 6 Universitat Pompeu Fabra (UPF), Barcelona, Spain; 7 Centro de Investigación Biomédica en Red de Enfermedades Raras (CIBERER), Spain; 8 Fondazione Stella Maris IRCCS, Pisa, Italy; 9 Department of Psychiatry, School of Medicine, and Department of Macromolecular. Science and Engineering, Case Western Reserve University, Cleveland, Ohio, USA; 10 Advocate Medical Group Adult Down Syndrome Center, Park Ridge, Illinois, USA; 11 Institut Jérôme Lejeune, Paris, France; 12 Department of Developmental Neuroscience, IRCCS Fondazione Stella Maris, Pisa, Italy; 13 Department of Clinical and Experimental Medicine, University of Pisa, Pisa, Italy; 14 Pediatric Unit, Bambino Gesù Children's Hospital, IRCCS, Rome, Italy; 15 Division of Pediatric Endocrinology, Saarland University Medical Center, Homburg/Saar, Germany; 16 Department of Neurology, Ludwig-Maximilians-Universität München, Munich, Germany; 17 German Center for Neurodegenerative Diseases, site Munich, Munich, Germany; 18 Munich Cluster for Systems Neurology (SyNergy), Munich, Germany; 19 Department of Population Policy and Practice, UCL Great Ormond Street Institute of Child Health, University College London, UK; 20 Whittington NHS Trust, London, UK; 21 Down Syndrome Medical Interest Group, London, UK; 22 Geriatric Department, Fondazione Policlinico Universitario A. Gemelli IRCCS, Rome, Italy; 23 Department of Human Genetics, School of Medicine, Emory University, Atlanta, Georgia, USA; 24 Institute of Psychiatry, Psychology, and Neuroscience, Department of Forensic and Neuro-developmental Sciences, King’s College London, London, UK; 25 The London Down Syndrome (LonDownS) Consortium, London, UK

## Abstract

**Background:**

People with Down syndrome (DS) are one of the highest risk groups for mortality associated with COVID-19, but outcomes may differ across countries due to different co-morbidity profiles, exposures, and societal practices, which could have implications for disease management. This study is designed to identify differences in clinical presentation, severity, and treatment of COVID-19 between India and several high-income countries (HICs).

**Methods:**

We used data from an international survey to examine the differences in disease manifestation and management for COVID-19 patients with DS from India vs HIC. De-identified survey data collected from April 2020 to August 2021 were analysed.

**Results:**

COVID-19 patients with DS from India were on average nine years younger than those from HICs. Comorbidities associated with a higher risk for severe COVID-19 were more frequent among the patients from India than from HICs. Hospitalizations were more frequent among patients from India as were COVID-19-related medical complications. Treatment strategies differed between India and HICs, with more frequent use of antibiotics in India. The average severity score of 3.31 was recorded for Indian DS in contrast to 2.3 for European and 2.04 for US cases.

**Conclusions:**

Presentation and outcomes of COVID-19 among individuals with DS were more severe for patients from India than for those from HIC. Global efforts should especially target vaccination campaigns and other risk-reducing interventions for individuals with DS from low-income countries.

## INTRODUCTION

The outbreak of SARS CoV-2 infection, leading to the pandemic of coronavirus disease 2019 (COVID-19), poses a serious threat to the health of individuals with intellectual disabilities, including those with Down syndrome (DS) [1-3]. DS, caused by the presence of an extra copy of chromosome 21, is the most frequent live-born genetic form of intellectual disability. It is characterized by many potential co-occurring health conditions, specific dysmorphic features, and dysregulated aspects of the immune system [4]. The cause of these medical conditions among individuals with DS is likely due to a gene dosage imbalance, owing to the triplication of chromosome 21 specific genes and their downstream effects on gene regulation [5]. At the immune system level, this genetic backdrop may lead to an altered immune response among individuals with DS. Altogether, these factors are likely to increase the vulnerability of individuals with DS to severe COVID-19 [6,7].

Comorbidities associated with DS may elevate the risk of severe respiratory tract infection, including SARS-CoV-2 [8]. To gain insight into the vulnerability, manifestation, and effects of COVID-19 on DS, the Trisomy 21 Research Society (T21RS) has conducted a global online survey of COVID-19 patients with DS since April 2020. The survey was designed to address issues related to the clinical manifestation and treatment of COVID-19 among people with DS and to identify risk factors of severity. The initial results [8] of the international survey based on >1000 individuals with DS suggested a more severe manifestation of SARS-CoV-2 infection with more severe medical complications and increased mortality among adults with DS compared to people without DS.

While the difference in the severity of COVID-19 between people with and without DS has been well established [8-11], our previous work suggested that there could be differences in health care resources, demographics, and culture between low- and high-income countries which could affect the treatment and outcome of COVID-19, particularly for high-risk groups like people with DS. We used data from the international T21RS online survey of COVID-19 patients with DS to investigate differences in COVID-19 severity and treatment between patients with DS from India (a lower-middle-income country (LMIC)) vs several high-income countries (HICs) in Europe and United States of America (USA).

## METHODS

### Trisomy 21 Research Society Down syndrome survey

An online survey was designed by the T21RS COVID-19 global task force in March 2020, to record de-identified epidemiological details of COVID-19 among individuals with DS [8]. Two parallel surveys were conducted, one for caregivers/family members of COVID-19-affected individuals with DS and another for clinicians. Both of these surveys included information related to existing health conditions and symptoms, treatments and outcomes, of COVID-19 among those with DS. The clinician survey recorded more detailed treatment strategies and COVID-19-related medical complications. To avoid replicating the entry of the same subject reported by both the caregiver and clinician from the same locality, we excluded duplicated entries based on identical age, gender, country, and other specific demographics. Although we used several criteria to identify false data entry, we could not eliminate the potential that caregivers may have been untruthful in their responses.

The survey was implemented through REDCap, a survey and database management system, and was hosted at Emory University. As we recorded data from the early phase of the COVID-19 pandemic, and as the diagnosis capacities differed between countries, we use the term “case” to refer to individuals with DS of all ages, who tested positive for SARS-CoV-2 infection or reported signs or symptoms of COVID-19. The survey was disseminated through clinical routes (eg, Down syndrome medical interest group and health service providers), Down syndrome associations in the USA, India, Spain, UK, France, Italy, Germany, Brazil, and Spanish-speaking Latin America, and DS registries (eg, NIH DS-Connect) as well as via the T21RS website. The present study includes the entries from April 8, 2020, to August 2, 2021.

### Ethics statement

Each institution that participated in this global study and disseminated the survey through families and clinicians obtained IRB/ethics approval (Table S1 in the **Online Supplementary Document**). The study was performed following the Declaration of Helsinki. All participants who completed the questionnaires provided informed consent.

### Statistical analysis

We included data on COVID-19 patients with DS that were recorded between April 8, 2020, and August 2, 2021. Only individuals with information on age and gender were included in the analyses. We categorized the countries of residence into LMICs vs HICs based on the World Bank income classification system [12]. We focused on India as the LMIC because of the limited number of participants from other LMICs. Countries classified as HIC included Argentina, Australia, Austria, Belgium, Canada, Cyprus, Denmark, Finland, France, Germany, Greece, Iceland, Ireland, Italy, Luxembourg, Malta, Netherlands, Norway, Portugal, Spain, Sweden, UK, and the USA. We used descriptive statistics to show the demographic detail, COVID-19 outcomes, and comorbidities of the participants. For the unadjusted comparison, Fisher exact test was used to investigate differences in the prevalence of comorbidities and medical complications between COVID-19 patients with DS from HIC vs India. Adjusted comparisons were conducted using Poisson regression with robust standard errors for the binary variables and using linear regression for the continuous variables. Comparisons of comorbidities were adjusted for gender, age, and survey (clinician vs · family survey) and comparison of medical complications and severity of disease were additionally adjusted for comorbidities associated with severe COVID-19. Comparison of medication used for patients from HIC vs India was adjusted for age, sex and the COVID-19 severity score. We characterized the severity of COVID-19 using attributes related to medical complications (presence of viral pneumonia, acute respiratory distress syndrome, and death) and the need for intervention (eg, hospitalization, mechanical ventilation support) using a composite severity score. The composite severity score proposed by Cao et al. [10] classifies COVID-19 patients based on the need for hospitalization and oxygen administration, resulting in the following seven category ordinal scale: 1-2 = not hospitalized; 3 = hospitalized, not requiring supplemental oxygen; 4 = hospitalized, requiring supplemental oxygen; 5 = hospitalized, requiring nasal high-flow oxygen therapy, non-invasive mechanical ventilation, or both; 6 = hospitalized, requiring invasive mechanical ventilation, extracorporeal membrane oxygenation (ECMO), or both; 7 = death. Due to our survey’s data limitations, we combined category 5 and 6 into one category in our analyses.

In a sensitivity analysis, we restricted the comparison between COVID-19 patients with DS from HIC vs India to those that were hospitalized, as these numbers might be less affected by selection bias.

The data analyses were done using R (version 4.0.0, Foundation for Statistical Computing, Vienna, Austria).

### Role of the funding sources

The funders helped in the dissemination of the online surveys and provided partial support for the biostatistician’s work. They had no role in the study design; collection, analysis, or interpretation of data, manuscript writing, or in the decision to submit the paper for publication.

## RESULTS

### Demographics

The distribution of demographic variables is presented in Table 1. The COVID-19 patients from India were on average younger than those from HIC. The mean age of the COVID-19 patients in India was 24.9 ± 12 · 88 (mean±SD) years, on average nine years younger than those from HIC (33.53 ± 19.07 years) and the difference was statistically significant (*P* < 0.0001). The gender distribution among the reported COVID-19 patients with DS from India and HIC was similar (India = 46.0% female, HIC = 44.7% female).

**Table 1:** Study characteristics of the T21RS COVID-19 patients with Down syndrome stratified by high-income countries (HICs) vs India*

	HIC	India	*P*-value†
**N**	794	478	
**Family survey, n (%)**	307 (38.7)	197 (41.2)	0.400
**Age, mean (SD)**	33.53 (19.07)	24.90 (12.88)	<0.001
**Gender, n (%)**			0.374
Female	355 (44.7)	220 (46.0)	
Male	436 (54.9)	258 (54.0)	
Other	3 (0.4)	0 (0.0)	
**Level of intellectual disability, n (%)**			<0.001
Borderline/normal/mild	126 (18.3)	47 (10.2)	
Moderate	404 (58.7)	331 (71.8)	
Severe/Profound	158 (23.0)	83 (18.0)	
**Living situation before the COVID-19 outbreak, n (%)**			<0.001
Living at home with family	425 (55.5)	410 (98.6)	
Living alone with support	10 (1.3)	1 (0.2)	
Small group home with support	149 (19.5)	4 (1.0)	
Residential care facility	181 (23.6)	1 (0.2)	
**Outcome of disease at last evaluation, n (%)**			<0.001
Not currently in hospital but with symptoms	59 (8.1)	92 (19.5)	
Currently in hospital with symptoms	15 (2.1)	123 (26.1)	
Tested positive but still no symptoms	48 (6.6)	12 (2.5)	
Recovered from COVID-19	506 (69.8)	197 (41.7)	
Died from complications due to COVID-19	97 (13.4)	48 (10.2)	
Medical complications due to COVID-19‡, n (%)	208 (43.5)	187 (67.0)	<0.001
Admitted to a hospital because of COVID-19, n (%)	300 (38.4)	344 (72.9)	<0.001
Days in hospital, mean (SD)	13.39 (18.99)	13 · 56 (3.96)	0.873
In an intensive care unit, n (%)	70 (24.8)	231 (67.2)	<0.001
Days in ICU, mean (SD)	13.07 (14.26)	7.66 (2.47)	<0.001

*Percentages were calculated after excluding missing values.

†Tested with χ^2^ test (categorical variables) or *t* test (continuous variables).

‡Question only part of the clinician survey and not the family survey.

The living situation of individuals with DS before the pandemic outbreak was significantly different (*P* < 0.001), where most COVID-19 patients with DS from India lived at home with their family (98.6%). In HICs, 55.5% of individuals with DS lived at home with their family, 23.6% lived in residential care facilities, and 19.5% in small group homes with support.

The distribution of the COVID-19 patients with DS among the different categories of intellectual disability also showed a significant difference (*P* <  · 001) with more patients from India being assigned to the category “moderate” in comparison to patients from HIC (**Table 1**).

### Comorbidities of COVID-19 patients with Down syndrome

We catalogued 20 different comorbidities common among people with DS [13-15]. We determined their frequency, stratified by country income level (HIC vs India, **Table 2**). We obtained the risk ratio (RR) of each comorbidity, using HIC as the reference category in the analyses, and adjusting for gender, age, and survey type (clinician vs family survey). We observed a significantly elevated frequency of comorbid conditions among COVID-19 patients with DS from India compared to those from HIC. Of the 20 conditions examined, the RR was significantly increased for 16 conditions (Table 2). For example, COVID-19 patients with DS from India had a 15 times increased risk of having diabetes (RR = 15.3, 95% CI = 10.3-22.9), 17 times increased risk of hypertension (RR = 17.2, 95% CI = 9.5-31.0) and 20 times increased risk of chronic liver disease (RR = 20.0, 95% CI = 11.3-35.3). A similar distribution of comorbidities was observed when restricting the comparison to those who were hospitalized with wide confidence intervals due to the smaller sample sizes (Table S2 in the **Online Supplementary Document**).

**Table 2:** Comparison of comorbidities in COVID-19 patients with Down syndrome from high-income countries (HIC) and India

	Unadjusted comparison				Adjusted comparison†		
	**HIC (N = 794)**	**India (N = 478)**	*P*-value*		**RR‡**	**95% CI**	***P*-value**
Obesity, n (%)	194 (27.8)	218 (52.2)	<0.001		2.14	(1.83-2.51)	<0.001
Alzheimer disease/dementia, n (%)	124 (17.8)	51 (12.1)	0.015		2.49	(1.80-3.43)	<0.001
Thyroid disorder, n (%)	339 (46.5)	223 (52.3)	0.063		1.35	(1.19-1.53)	<0.001
Seizures/epilepsy, n (%)	88 (12.6)	157 (37.1)	<0.001		4.69	(3.70-5.94)	<0.001
Blood cancer, n (%)	2 (0.3)	10 (2.4)	0.003		13.9	(1.29-148)	0.030
Other cancer, n (%)	1 (0.1)	2 (0.5)	0.645		9.35	(0.05-1760)	0.403
Immuno-compromised, n (%)	17 (2.4)	25 (6.1)	0.003		2.26	(1.14-4.50)	0.020
Obstructive sleep apnea, n (%)	205 (29.7)	178 (42.4)	<0.001		1.42	(1.21-1.68)	<0.001
Hypertension, n (%)	18 (2.6)	103 (24.6)	<0.001		17.2	(9.53-31.0)	<0.001
Diabetes, n (%)	28 (4.0)	173 (41.1)	<0.001		15.3	(10.3-22.9)	<0.001
Cerebrovascular disease, n (%)	12 (1.7)	4 (1.0)	0.443		0.551	(0.18-1.71)	0.301
Coronary heart disease, n (%)	18 (2.6)	48 (11.4)	<0.001		4.66	(2.71-8.04)	<0.001
Chronic renal disease, n (%)	26 (3.7)	53 (13.0)	<0.001		4.06	(2.62-6.28)	<0.001
Chronic liver disease, n (%)	12 (1.7)	149 (36.3)	<0.001		20.0	(11.3-35.3)	<0.001
Chronic lung disease, n (%)	60 (8.5)	230 (54.8)	<0.001		6.00	(4.58-7.87)	<0.001
Celiac disease, n (%)	45 (6.4)	25 (6.2)	1		0.840	(0.53-1.34)	0.467
Gastroesophageal reflux, n (%)	97 (13.7)	123 (29.6)	<0.001		2.18	(1.70-2.80)	<0.001
Irritable bowel syndrome, n (%)	14 (2.0)	97 (23.4)	<0.001		10.6	(6.18-18.0)	<0.001
Hepatitis B infection, n (%)	12 (1.8)	3 (0.7)	0.268		1.01	(0.26-3.96)	0.991
Congenital heart defect, n (%)	235 (31.0)	219 (50.9)	<0.001		1.46	(1.26-1.68)	<0.001

HIC – high-income country

*Fisher exact test.

†Poisson regression with robust standard errors adjusted for gender, age, and survey (clinician vs family survey).

‡HIC as reference category.

### Hospitalization and outcome of COVID-19 at last evaluation

COVID-19 patients with DS from India were more often admitted to hospital (India = 72.9% vs HIC = 38.4%) and to an intensive care unit (ICU) (India = 67.2% vs HIC = 24.8%) (**Table 3**). Even after adjusting for differences in age, gender, survey type (clinician vs family survey) and comorbidities between the two groups, COVID-19 patients with DS from India were more than twice as likely to be hospitalized and admitted to an ICU than those from HIC (hospitalization: RR = 2.20, 95% CI = 1.67, 2.90; ICU: RR = 2.35, 95% CI = 1.54, 3.68).

**Table 3:** Comparison of medical complications and indicators for severity of disease in COVID-19 patients with Down syndrome from high-income countries (HIC) and India

	Unadjusted comparison						Adjusted comparison†				
	**HIC**	**N**	**India**	**N**	*P*-value*		**Beta‡**	**RR§**	**95% CI**	***P*-value**
Acute respiratory distress syndrome, n (%)	80 (47.6)	168	124 (67.8)	183	<0.001		N/A	1.44	(0.79, 2.64)	0.0783
Viral pneumonia associated with COVID-19, n (%)	187 (95.4)	196	33 (20.1)	164	<0.001		N/A	0.257	(0.13, 0.48)	<0.001
Mechanical ventilation, n (%)	58 (11.7)	494	161 (41.8)	385	<0.001		N/A	2.83	(1.68, 4.83)	<0.001
Hospitalization, n (%)	300 (38.4)	782	344 (72.9)	472	<0.001		N/A	2.20	(1.67, 2.90)	<0.001
ICU admission (among those who were hospitalized), n (%)	70 (24.8)	282	231 (67.2)	344	<0.001		N/A	2.35	(1.53, 3.68)	<0.001
Death, n (%)	97 (12.9)	754	48 (10.1)	473	0.179		N/A	1.52	(0.65, 3.42)	0.318
Oxygen therapy, n (%)	180 (44.7)	403	205 (94.0)	218	<0.001		N/A	2.98	2.07, 4.31)	<0.001
CPAP/BIPAP, n (%)	13 (6.1)	212	6 (3.0)	203	0.189		N/A	1.25	(0.46, 3.40)	0.66
Extracorporeal membrane oxygenation¶, n (%)	0 (0.0)	374	3 (1.5)	205	0.082		N/A	N/A	(N/A, N/A)	N/A
Continuous renal-replacement therapy¶, n (%)	3 (0.8)	376	37 (17.8)	208	<0.001		N/A	N/A	(N/A, N/A)	N/A
Severity score**, mean (SD)	2. 21 (1.77)	783	3.31 (1.65)	477	<0.001		1.2	N/A	(0.84, 1.4)	<0.001

HIC – high-income country, N/A – not applicable, RR – risk ratio

*Fisher exact test.

†Independent variable: country of residence (India vs HIC, HIC as reference category); dependent variable: indicators of COVID-19 severity; adjusted for survey (clinician vs · family survey), gender, age, Alzheimer disease/dementia, obesity, thyroid disorder, seizures/epilepsy, obstructive sleep apnea, hypertension, diabetes, gastroesophageal reflux, congenital heart defect.

‡Beta estimate from linear regression model with the severity score as dependent variable.

§RR from Poisson regression with robust standard errors.

Questions only part of the clinician survey.

¶No adjusted comparison because of too few cases.

**See “statistical analysis” section for details on the definition of severity score, defined according to Cao et al. [10].

COVID-19 patients with DS from India were also about three times as likely to be treated with oxygen/ventilation support than those from HIC, including mechanical ventilation (RR = 2.83, 95% CI = 1.68-4.83) and oxygen therapy (RR = 2.98, 95% CI = 2.07-4.31). However, these associations were attenuated and only significant for mechanical ventilation when restricting the analyses to the COVID-19 patients who were hospitalized (mechanical ventilation: RR = 1.77, 95% CI = 1.06-2.99; oxygen therapy: RR = 1.09, 95% CI = 0.73-1.61); Table S3 in the **Online Supplementary Document**).

Consistent with this, COVID-19 patients with DS from India had a 1.2 points higher severity score (95% CI = 0.84-1.4) than those from HIC after adjusting for differences in age, gender, survey type (clinician vs family survey), and comorbidities between the two groups.

Based on clinician reports, viral pneumonia was more common among COVID-19 patients with DS from HIC (HIC = 95.4%, India = 20.1%) and this difference was robust to adjustment for confounding (*P* < 0.001). Acute respiratory distress syndrome (ARDS) was more commonly reported by clinicians among COVID-19 patients with DS from India than among those from HIC (RR = 1.44, 95% CI = 0.79- 2.64), though the difference was not statistically significant in adjusted comparison. Mortality rates, reported in both family and clinician surveys, were higher among patients from India compared with HIC, but these differences were not statistically significant (RR = 1.52, 95% CI = 0.65-3.42).

### Treatment

The medications for COVID-19-infected DS individuals were reported only in the clinician surveys. As shown in **Table 4**, the use of specific medication strategies varied in frequency between India and HIC. After adjusting for group differences in age, sex, and severity score, patients from India were more likely to be treated with azithromycin (RR = 1.64, 95% CI = 1.37-1.9), chloroquine (RR = 3.04, 95% CI = 1.04-8.87), hydroxychloroquine (RR = 1.36, 95% CI = 1.14-1.63), and remdesivir (RR = 6.9, 95% CI = 3.33-14.3) (**Table 4**). On the contrary, antibiotics other than azithromycin (RR = 0.79, 95% CI = 0.67-0.93), systemic glucocorticoid (RR = 0.56, 95% CI = 0.41-0.77), and low molecular weight heparin-prophylactic dose (RR = 0.11, 95% CI = 0.06-0.22) were used more frequently in HIC than in India (**Table 4**). The observed differences in the use of medications were similar when restricting the analyses to those COVID-19 patients with DS who were hospitalized (Table S4 in the **Online Supplementary Document**).

**Table 4:** Comparison of use of medication for COVID-19 patients with Down syndrome from high-income countries (HIC) and India

	Unadjusted comparison					Adjusted comparison†		
	**HIC**	**N**	**India**	**N**	*P*-value*	**RR‡**	**95% CI**	***P*-value**
Are/was the person treated with medications for COVID-19	182 (44.6)	408	220 (100.0)	220	<0.001	1.64	(1.46, 1.84)	<0.001
Azithromycin	79 (53.0)	149	200 (94.3)	212	<0.001	1.64	(1.37, 1.95)	<0.001
Other antibiotics (oral or IV)	118 (72.0)	164	96 (46.2)	208	<0.001	0.79	(0.67, 0.93)	0.004
Chloroquine	5 (3.5)	143	27 (14.9)	181	0.001	3.04	(1.04, 8.87)	0.042
Hydroxychloroquine	85 (54.8)	155	204 (95.3)	214	<0.001	1.36	(1.14, 1.63)	<0.001
Remdesivir	7 (11.9)	59	178 (89.0)	200	<0.001	6.9	(3.33, 14.3)	<0.001
Other antiviral agents	8 (13.3)	60	17 (10.1)	169	0.647	1.39	(0.636, 3.06)	0.410
Systemic glucocorticoids	67 (45.6)	147	54 (27.6)	196	0.001	0.56	(0.41, 0.77)	<0.001
Tocilizumab	12 (8.3)	144	88 (44.2)	199	<0.001	3.98	(2.15, 7.34)	<0.001
Antifungal medication	5 (3.5)	144	2 (1.1)	179	0.289	0.10	(0.02, 0.50)	0.006
Low molecular weight heparins (prophylactic dose)	23 (40.4)	57	11 (5.7)	192	<0.001	0.11	(0.06, 0.22)	<0.001
Low molecular weight heparins (therapeutic dose)	10 (17.5)	57	56 (28.3)	198	0.144	1.4	(0.69, 2.85)	0.360
Other anticoagulants (oral or IV)	2 (3.5)	57	33 (17.3)	191	0.016	6.97	(1.05, 46.2)	0.044
Colchicine	2 (1.4)	141	2 (1.0)	195	1	6.73	(1.12, 40.6)	0.037
Melatonin	6 (4.3)	139	24 (12.2)	197	0.022	2.65	(0.96, 7.33)	0.060

HIC – high-income country, RR – risk ratio

*Fisher exact test.

†Independent variable: country of residence (India vs HIC, HIC as reference category); dependent variable: medication; adjusted for age, sex, severity score (not adjusted for type of survey because questions on medication were only part of the clinician survey).

‡RR from Poisson regression with robust standard errors.

### Incidence and severity of the cases over time

**Figure 1:**
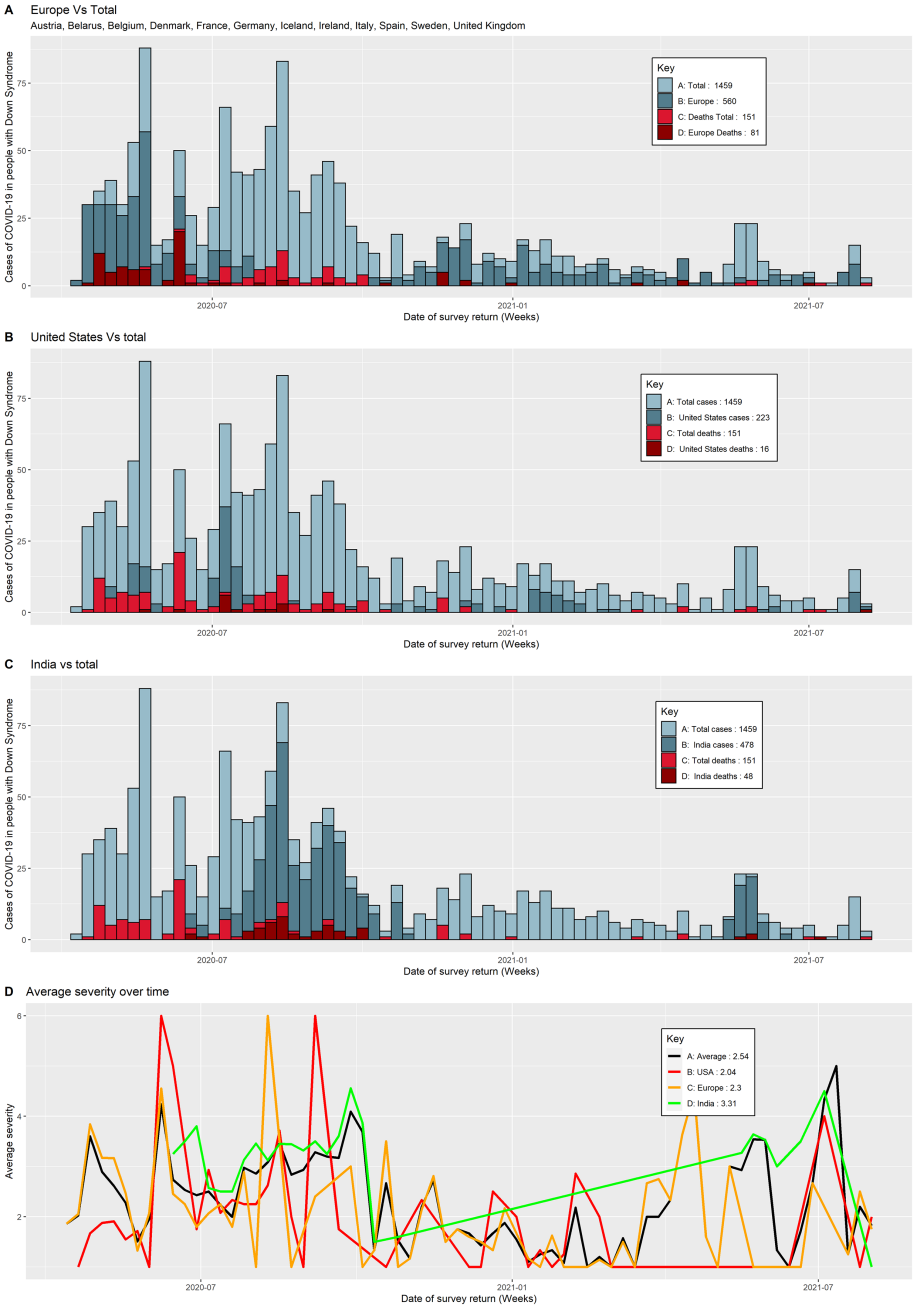
Distribution of COVID-19 patients with Down syndrome over time (time at which they were entered into the survey). **Panel A.** Europe vs all. **Panel B.** USA vs all, **Panel C.** India vs all, **Panel D.** Severity score over time stratified by Europe, USA, India.

To examine whether the pandemic phase may explain some of the differences observed between India and HICs, we compared the dates on which the surveys were completed by COVID-19 positive respondents from specific countries of residence (USA, Europe, and India), stratifying by country, to completion dates of all surveys. For Europe, the initial peak of participation was during May-June 2020 and most deaths occurred during this time period. Secondary peaks were also observed in December 2020 and January 2021. After that, a moderate number of cases were recorded consistently until August 2021 (**Figure 1**, Panel A). The pattern observed for the cases from the USA was similar to Europe but shifted to a later time period: the initial peak was in July 2021, again with a consequent increase in deaths; a second peak was shifted to January-February 2021 (**Figure 1**, Panel B).

The completed surveys from India showed a different pattern compared with Europe and USA (**Figure 1**, Panel C). During the first wave of the pandemic, most of the Indian cases were recorded from August to October 2020, and became more frequent during the second pandemic wave, from February to April 2021. Out of a total of 48 death cases reported from India, most of the entries were recorded during the first wave, August to October 2020 (**Figure 1**, Panel C).

We calculated the average severity score of 2.54 for all 1459 reported DS cases over time (Europe = 2.3, USA = 2.04, and 3.31 for the India = 3.31; **Figure 1**, Panel D). In Europe and the USA, the average severity score of the study participants was largest at the beginning of the pandemic and smaller during later waves of the pandemic. We did not see a similar trend for COVID-19 patients from India, where the average severity score did not decrease over time.

## DISCUSSION

In this international survey of 1272 COVID-19 patients with DS, we showed that patients from India (N = 478), which is classified as an LMIC according to the World Bank classification [12], were more severely ill from COVID-19 than patients from HICs from Europe and USA (N = 794). COVID-19 patients with DS from India were on average nine years younger than those from HICs, but they had significantly more comorbidities, increasing their risk for severe COVID-19, as seen by higher rates of medical complications and more invasive treatment strategies used. Even after adjusting for these group differences in age and comorbidities, admission rates to hospital and ICU as well as invasive treatment strategies were still more common among COVID-19 patients from India than among those from HIC, indicating that they were more severely ill at the time they were admitted to hospital than their counterparts from HIC.

The average severity score over time for COVID-19 patients with DS in India was higher than for those from HIC and did not decrease over time as it did for patients from Europe and USA. This indicates that mitigation and treatment strategies led to a decrease of severely ill COVID-19 patients in Europe and the USA over time but did not do so to the same degree in India. There is some anecdotal evidence of instances in which life-support to severely ill patients with DS may have been selectively withdrawn, due to limited availability of health care resources (rationing) during the peak COVID-19 pandemic wave in that country. However, hard evidence of such events may never come to light.

The Indian COVID-19 patients with DS whose data were analysed in the present study were, on average, nine years younger than those from HIC. This may be due to cultural differences in the acceptance and inclusion of adults with DS into families or overall society. Although health care and access to early education for neonates and young children with DS have improved considerably in India over the past few decades, many adults with DS still receive less care (than other healthy family members) by their families, and public and private support for their training and inclusion in society are still at very low levels. This situation may have led to less participation of families with adults with DS compared with families with children with DS. This participation bias may have also led to an underestimation of the frequency of severe COVID-19 outcomes in India, given that lack of support for adults with DS would increase their risk for social and health disparities and consequently their risk for severe COVID-19. We also noticed that the living situation of people with DS in the Indian sample was markedly different than for those in HICs. Nearly 98% of all Indian patients were reported to live at home with family members, while in HICS, 19% lived in small group homes and 23% in residential care facilities. This difference probably reflects a mixture of cultural issues, lack of dedicated living facilities for people with DS and other intellectual disabilities in India, and lack of financial resources.

We observed a significantly elevated frequency of comorbid conditions among COVID-19 patients with DS from India in comparison to those from HICs. From the 20 conditions examined, the RR was significantly increased for 16 conditions (**Table 2**), many of them being known risk factors for severe COVID-19 and death [16,17]. However, we did not find any published literature on the difference in the prevalence of comorbid conditions among the general population with DS from India and HIC. We speculate that the observed medical vulnerability may stem from the average lower socio-economic condition of the Indian sample, lack of strong social support systems, and/or lack of enough professionals who are trained to address specific health care needs of individuals with DS. Other explanations to justify the observed difference in the frequency of co-morbid conditions between India and HIC may include differential reporting, differential treatment leading to persistent symptoms and a higher frequency of associated risk factors for the Indian subjects.

COVID-19 patients with DS from India were more often admitted to hospital (India = 72 · 9% vs HICs = 38.4%) and to an intensive care unit (ICU) (India = 67 · 2% vs HICs = 24 · 8%). Furthermore, hospitalised COVID-19 patients with DS from India were more severely ill than their counterparts from HIC, indicating that mild and early stages of COVID-19 diseases were often overlooked in India due to a lack of testing capacity. This observation is particularly important for high-risk groups like people with DS, for whom an early intervention could make a significant difference in terms of disease progression. However, lack of treatment at home at the beginning of the first pandemic wave in India may also have contributed to this outcome. During the first wave of the COVID-19 pandemic, India suffered from a shortage of ventilators and oxygen supply in the face of heavy demand. However, we did not observe any significant differences overall in mortality rates between DS patients from India and HIC. Possible explanations include: 1) non-participation of older adults with DS, the most vulnerable group; 2) non-participation of families who experienced a death of their loved one with DS; or 3) under-representation of patients from the lowest socio-economic groups in India who may have been at increased risk for poor outcomes.

We observed large differences between the medications that were used for treating COVID-19 among people with DS from India and HIC. Azithromycin, chloroquine, hydroxychloroquine, remdesivir and melatonin were used more frequently in India, while antibiotics besides azithromycin, systemic glucocorticoid, and low molecular weight heparin-prophylactic dose were used more frequently in HICs. This difference in treatment strategy may reflect the varying severity of disease manifestation among the individuals with DS from India and HICs. However, as these differences in the use of medication were still significant after adjusting for age, sex and severity score, they more likely reflect the differences in health care practices across the countries. Most patients from India were recorded during the first pandemic wave, when there was limited knowledge about the effectiveness of specific drugs. For example, chloroquine and hydroxychloroquine were used frequently in the early phase of the first wave, but proven later ineffective and were not used in the second wave. In contrast, the treatment regime in Europe and USA was more conservative and included steroids, an effective medication with few side effects. This might have contributed to a less severe course in cases from these countries.

Our study has some potential limitations. We selected India as the representative for LMICs, but other country-specific differences might exist between LMICs, which should be investigated in future studies. Similarly, country-specific differences in health care system may influence the observed differences in the data. Moreover, diagnostic techniques between India and HICs may differ, which could explain differences in the frequency of medical complications like viral pneumonia. We could not rule out the possibility of suspicious entries, though we have taken several measures of data cleaning. Most entries were recorded during the first wave; cases from the second wave may be under-represented. We may have oversampled financially stable families in India compared with underprivileged families or those with less access to internet facilities from rural areas, which most likely affected the results. However, this may also be true for HIC.

## CONCLUSIONS

Our study revealed for the first time that the severity of COVID-19 and its management among people with DS differed by income level of the country (India vs HICs) Global efforts should target vaccination campaigns and other risk-reducing interventions to individuals with DS from LMICs, as limited resources increase their risk for severe outcomes of disease.

## Additional material


Online Supplementary Document
