# Supplementary material for: Differences in clinical presentation, severity, and treatment of COVID-19 among individuals with Down syndrome from India and high-income countries: Data from the Trisomy 21 Research Society survey
Source: J Glob Health. 2022 Aug 8;12:05035. doi: 10.7189/jogh.12.05035 (PMC9356581; doi:10.7189/jogh.12.05035)
Supplement: Online Supplementary Document [file jogh-12-05035-s001.pdf]

Table S1. Each institution that planned to disseminate the survey obtained IRB/ethics approval

**Spain**	The study was approved by the Hospital del Mar ethics committee (CEIC Parc de Salut Mar, CEim 2020/9197)
**United Kingdom**	The T21RS survey was approved by the Health research agency (HRA) 20/HRA/2452. The ISARIC4C analysis was approved by the study board (IDAMAC)
**Brazil**	This study was approved by the Brazilian Federal Ethics Committee (CONEP, CAAE: 30847520.8.0000.0071)
**Emory University, U.S.A.**	This study was deemed exempt from human subjects research under 45 CFR 46.104(d)(2i) (IRB ID: STUDY00000386)
**Italy**	The study was approved by the Bambino Gesù children's Hospital Ethics Committee (2091_OPBG_2020)
**Advocate Health Care Institutional Review Board**	Determined to have Exempt Status IRB# 20-151ET
**France**	This study was approved by CPP Sud Mediterranée IV dated 29/04/2020 (ID RCB 2020-A00940-39)
**Ludwig-Maximilians-Universität (LMU), Munich, Germany**	Determined to have Exempt Status (IRB ID: 20-573 KB)
**India**	The study was approved by the Ethics Committee constituted by the University of Calcutta (CU/BIOETHICS/HUMAN/2306/3044/2020)

Table S2. Comparison of comorbidities in hospitalized COVID-19 patients with Down syndrome from high-income countries (HIC) and India

	Unadjusted comparison			Adjusted Comparison†		
	**HIC (N=300)**	**India (N=344)**	***P*-value***	**RR‡**	**95% CI**	***P*-value**
Obesity, n (%)	92 (35.4)	167 (56.8)	<0.001	1.66	(1.35, 2.05)	<0.001
Alzheimer disease/dementia, n (%)	73 (27.4)	48 (16.3)	0.002	2.35	(1.63, 3.39)	<0.001
Thyroid disorder, n (%)	138 (50.4)	175 (58.5)	0.061	1.58	(1.34, 1.88)	<0.001
Seizures/epilepsy, n (%)	43 (16.2)	129 (43.6)	<0.001	3.85	(2.78, 5.32)	<0.001
Blood cancer, n (%)	2 (0.8)	10 (3.4)	0.059	6.23	(0.49, 79.1)	0.16
Other cancer, n (%)	1 (0.4)	2 (0.7)	1	4.43	(0.02, 1120)	0.6
Immuno-compromised, n (%)	6 (2.3)	20 (7.0)	0.015	3.11	(0.88, 11)	0.08
Obstructive sleep apnea, n (%)	77 (30.0)	125 (42.4)	0.003	1.38	(1.08, 1.76)	0.01
Hypertension, n (%)	3 (1.1)	89 (30.0)	<0.001	60	(18.8, 192)	<0.001
Diabetes, n (%)	17 (6.5)	144 (48.8)	<0.001	11.3	(6.92, 18.5)	<0.001
Cerebrovascular disease, n (%)	4 (1.5)	4 (1.4)	1	0.814	(0.25, 2.7)	0.74
Coronary heart disease, n (%)	5 (1.9)	41 (14.0)	<0.001	6.59	(2.64, 16.4)	<0.001
Chronic renal disease, n (%)	13 (4.9)	44 (15.4)	<0.001	3.01	(1.65, 5.47)	<0.001
Chronic liver disease, n (%)	4 (1.5)	98 (34.0)	<0.001	20.5	(7.54, 55.5)	<0.001
Chronic lung disease, n (%)	28 (10.4)	149 (50.3)	<0.001	4.83	(3.19, 7.32)	<0.001
Celiac disease, n (%)	13 (4.9)	12 (4.3)	0.896	0.673	(0.30, 1.51)	0.34
Gastroesophageal reflux, n (%)	32 (12.0)	89 (30.6)	<0.001	2.67	(1.80, 3.98)	<0.001
Irritable bowel syndrome, n (%)	7 (2.7)	71 (24.3)	<0.001	7.32	(3.43, 15.6)	<0.001
Hepatitis B infection, n (%)	4 (1.6)	3 (1.1)	0.898	1.08	(0.15, 7.76)	0.94
Congenital heart defect, n (%)	77 (27.7)	169 (56.0)	<0.001	1.69	(1.35, 2.11)	<0.001

*Fisher's exact test
†Poisson regression with robust standard errors adjusted for gender, age, and survey (clinician vs· family survey)
‡HIC as reference category

Table S3: Comparison of medical complications and severity of disease in hospitalized COVID-19 patients with Down syndrome from high-income countries (HIC) and India

	Unadjusted comparison					Adjusted comparison†			
	**HIC**	**N**	**India**	**N**	***P*-value***	**Beta‡**	**RR§**	**95% CI**	***P*-value**
Acute respiratory distress syndrome‖, n (%)	75 (49.7)	151	121 (69.1)	175	0.001	N/A	1.44	(0.79, 2.64)	0.23
Viral pneumonia associated with COVID-19‖, n (%)	169 (97.1)	174	29 (18.5)	157	<0.001	N/A	0.22	(0.11, 0.43)	<0.001
Mechanical ventilation, n (%)	58 (22.4)	259	161 (47.9)	336	<0.001	N/A	1.77	(1.06, 2.99)	0.030
ICU admission (among those who were hospitalized), n (%)	70 (24.8)	282	231 (67.2)	344	<0.001	N/A	2.35	(1.52, 3.65)	<0.001
Death, n (%)	80 (28.2)	284	47 (13.8)	341	<0.001	N/A	0.96	(0.39, 2.35)	>0.9
Oxygen therapy‖, n (%)	169 (86.2)	196	213 (95.9)	222	0.001	N/A	1.09	(0.73, 1.61)	0.70
CPAP/BIPAP‖, n (%)	11 (13.9)	79	16 (7.6)	211	0.153	N/A	0.73	(0.26, 2.06)	0.50
Continuous renal-replacement therapy‖, n (%)	3 (1.8)	167	40 (18.5)	216	<0.001	N/A	146	(13.3, 1610)	<0.001

*Fisher's exact test; †Independent variable: country of residence (India versus HIC, HIC as reference category); dependent variable: indicators of COVID-19 severity; adjusted for survey (clinician vs· family survey), gender, age, Alzheimer's disease/dementia, obesity, thyroid disorder, seizures/epilepsy, obstructive sleep apnea, hypertension, diabetes, gastroesophageal reflux, congenital heart defect ; ‡Beta estimate from linear regression model with the severity score as dependent variable; §RR from Poisson regression with robust standard error; ‖questions only part of the clinician survey; [6]no adjusted comparison because of too few cases

Table S4. Comparison of use of medication for hospitalized COVID-19 patients with Down syndrome from high-income countries (HIC) and India

	Unadjusted comparison					Adjusted comparison†		
	**HIC**	**N**	**India**	**N**	***P*-value***	**RR‡**	**95% CI**	***P*-value**
Are/was the person treated with medications for COVID-19	155 (87.6)	179	224 (100.0)	224	<0.001	1.12	(1.05, 1.19)	<0.001
Azithromycin	67 (53.6)	125	188 (89.1)	211	<0.001	1.6	(1.32, 1.92)	<0.001
Other antibiotics (oral or IV)	117 (83.0)	141	123 (57.2)	215	<0.001	0.703	(0.60, 0.82)	<0.001
Chloroquine	5 (4.2)	119	25 (13.1)	191	0.017	3.11	(1.02, 9.47)	0.046
Hydroxychloroquine	83 (62.9)	132	176 (80.7)	218	<0.001	1.19	(1, 1.41)	0.044
Remdesivir	7 (13.0)	54	172 (83.5)	206	<0.001	5.79	(2.85, 11.8)	<0.001
Other antiviral agents	8 (14.5)	55	29 (16.1)	180	0.946	1.4	(0.63, 3.15)	0.41
Systemic glucocorticoids	68 (54.8)	124	59 (28.9)	204	<0.001	0.495	(0.36, 0.67)	<0.001
IV immune globulin	6 (5.1)	118	108 (51.4)	210	<0.001	10.6	(4.05, 27.5)	<0.001
Tocilizumab	12 (9.9)	121	89 (42.8)	208	<0.001	3.71	(2.02, 6.84)	<0.001
Antifungal medication	5 (4.1)	121	2 (1.1)	182	0.179	0.101	(0.02, 0.49)	0.005
Low molecular weight heparins (prophilactic dose)	26 (50.0)	52	13 (6.5)	200	<0.001	0.121	(0.06, 0.23)	<0.001
Low molecular weight heparins (therapeutic dose)	11 (21.6)	51	58 (28.3)	205	0.428	1.42	(0.72, 2.82)	0.311
Other anti-coagulants (oral or IV)	2 (3.8)	53	33 (16.6)	199	0.033	6.47	(1.03, 40.7)	0.046
Colchicine§	0 (0.0)	118	2 (1.0)	200	0.722	N/A	N/A	N/A
Melatonin	3 (2.6)	115	30 (14.7)	204	0.001	4.52	(1.18, 17.3)	0.027

*Fisher's exact test; †Independent variable: country of residence (India versus HIC, HIC as reference category); dependent variable: medication; adjusted for age, sex, severity score (not adjusted for type of survey because questions on medication were only part of the clinician survey); ‡RR from Poisson regression with robust standard errors; §no adjusted comparison because of too few cases
